# Global burden of cerebral small vessel disease determined from large MRI studies: A systematic review and meta-analysis

**DOI:** 10.1177/17474930261441916

**Published:** 2026-04-04

**Authors:** Bonnie Yin Ka Lam, Yuan Cai, Huijing Zheng, Jize Wei, Rufus Akinyemi, Geert Jan Biessels, Hilde van den Brink, Christopher Chen, Marco Duering, Deborah Gustafson, Saima Hilal, Vincent Ming Ho Hui, Rajesh Kalaria, SangYun Kim, Maggie Li Man Lam, Frank-Erik de Leeuw, Ami Sin Man Li, Hugh S Markus, Anna Marseglia, John O’Brien, Leonardo Pantoni, Perminder S Sachdev, Eric E Smith, Joanna M Wardlaw, Adrian Wong, Ho Ko, Vincent Chung Tong Mok

**Affiliations:** 1 Division of Neurology, Department of Medicine and Therapeutics, The Chinese University of Hong Kong, Shatin, Hong Kong; 2 Gerald Choa Neuroscience Institute, Margaret K.L. Cheung Research Centre for Management of Parkinsonism and Lau Tat-cheun Research Center of Brain Degenerative Diseases in Chinese, Therese Pei Fong Chow Research Centre for Prevention of Dementia, Lui Che Woo Institute of Innovative Medicine, Li Ka Shing Institute of Health Science, The Chinese University of Hong Kong, Shatin, Hong Kong; 3 Neuroscience and Ageing Research Unit, Institute for Advanced Medical Research and Training, College of Medicine, University of Ibadan, Ibadan, Nigeria; 4 Centre for Genomic and Precision Medicine, College of Medicine, University of Ibadan, Ibadan, Nigeria; 5 Department of Neurology, University College Hospital, Ibadan, Nigeria; 6 Translational and Clinical Research Institute, Newcastle University, Newcastle upon Tyne, UK; 7 Department of Neurology and Neurosurgery, UMC Utrecht Brain Center, University Medical Center Utrecht, Utrecht, The Netherlands; 8 J. Philip Kistler Stroke Research Center, Department of Neurology, Massachusetts General Hospital, Harvard Medical School, Boston, MA, USA; 9 Memory Aging and Cognition Centre, Department of Pharmacology, Yong Loo Lin School of Medicine, National University of Singapore, Singapore; 10 Antaros Medical AB, Mölndal, Sweden; 11 Institute for Stroke and Dementia Research (ISD), University Hospital, LMU Munich, Munich, Germany; 12 Section for NeuroEpidemiology, Department of Neurology, State University of New York Downstate Health Sciences University, Brooklyn, NY, USA; 13 Memory, Aging and Cognition Center, National University Health System, Singapore; 14 Saw Swee Hock School of Public Health, National University of Singapore and National University Health System, Singapore; 15 Department of Neurology, Seoul National University College of Medicine and Clinical Neuroscience Center, Seoul National University Bundang Hospital, Seoul, South Korea; 16 Department of Neurology, Donders Institute for Brain Cognition and Behaviour, Radboudumc, Nijmegen, The Netherlands; 17 Stroke Research Group, Department of Clinical Neurosciences, University of Cambridge, Cambridge, UK; 18 Division of Clinical Geriatrics, Centre for Alzheimer Research, Department of Neurobiology, Care Sciences and Society, Karolinska Institutet, Stockholm, Sweden; 19 Department of Psychiatry, School of Clinical Medicine, University of Cambridge, Cambridge, UK; 20 Department of Biomedical and Clinical Sciences, University of Milan, Milan, Italy; 21 Department of Neurorehabilitation Sciences, Casa di Cura Igea, Milan, Italy; 22 Centre for Healthy Brain Ageing and School of Clinical Medicine, The University of New South Wales, Sydney, NSW, Australia; 23 Neuropsychiatric Institute, School of Psychiatry, The University of New South Wales, Sydney, NSW, Australia; 24 Division of Neurology, Department of Clinical Neurosciences and Hotchkiss Brain Institute, University of Calgary, Calgary, AB, Canada; 25 Centre for Clinical Brain Sciences, Edinburgh Imaging, Institute of Neuroscience and Cardiovascular Research, and UK Dementia Research Institute, University of Edinburgh, Edinburgh, UK

**Keywords:** Cerebral small vessel disease, prevalence, meta-analysis, white matter hyperintensities, lacunes, cerebral microbleed, perivascular space

## Abstract

**Background:**

Cerebral small vessel disease (SVD) is a major cause of ischemic stroke, intracerebral hemorrhage, and dementia. Despite its importance, there are few studies of its prevalence and how cerebral SVD varies across the world, different age ranges, sexes, and magnetic resonance imaging (MRI) parameters. SVD can be estimated using MRI neuroimaging markers, including white matter hyperintensities (WMHs), lacunes, cerebral microbleeds (CMBs), and perivascular spaces (PVS).

**Aims:**

This study aimed to document the global prevalence of SVD based on population-based or large community-based MRI studies and to determine how SVD prevalence varies by region, mean age, and sex. With SVD neuroimaging markers being the standard to assess SVD prevalence, we aimed to investigate how different MRI acquisition parameters may influence its prevalence.

**Summary of review:**

In this systematic review and meta-analysis, articles were searched from the Ovid MEDLINE and EMBASE databases between 1 January 2000 and 31 March 2024, without language restrictions. Title and abstract screening, full-text review, and data extraction were performed by at least two independent reviewers. The prevalence of SVD, subject demographic information, and MRI acquisition parameters were extracted. The Risk of Bias for Non-randomized Studies tool was used. The protocol was registered on PROSPERO (CRD42022311133). Of 14,582 studies identified, 246 studies spanning 40 countries were included in the systematic review. In the meta-analysis, 85 studies (88 cohorts) from 17 regions (n = 1,562,765) were included. The quality of studies was high (mean score 7.67 out of 8, ranging between 5 and 8). The pooled prevalence of moderate-to-severe WMH was 18.9%, and the pooled mean of WMH volume was 4.4 mL. Pooled prevalences of lacunes, cerebral microbleeds (CMBs), and moderate-to-severe perivascular spaces (PVS) were 11.2%, 10.3%, and 22.6%, respectively. A lower lacune prevalence (7.3% vs 13.3%; adjusted OR (aOR) [95% confidence interval (CI)]: 0.45 [0.30–0.68]) but higher PVS prevalence (30.9% vs 19.6%; aOR [95% CI]: 12.15 [2.12–69.46]) was found in Europe compared with Asia. A higher mean age of the studies was associated with a higher prevalence of most SVD markers, except for PVS. There was an overall trend of more lacunes and CMBs in males. MRI field strength, sequence used, and slice thickness could potentially influence the reported SVD prevalence, especially for WMH volume and CMB count. There was high heterogeneity in the studies (>95%) that was not resolved by performing analyses stratified by Global Burden of Disease (GBD) regions, age groups, study design, or MRI parameters.

**Conclusion:**

This systematic review and meta-analysis based on large MRI studies demonstrated that SVD is a common health problem affecting about one-fifth of the adult population. SVD prevalence differs in regions separated by geographical regions. SVD prevalence is higher with increasing age. There is an overall trend of more lacunes and CMBs in males. WMH volume and CMB are SVD markers prone to the variability of MRI acquisition parameters, and a harmonised SVD scanning protocol should be used. More studies from middle- and low-income regions would benefit the estimation of a truly global prevalence of SVD.

## Graphical abstract

**Graphical abstract:**
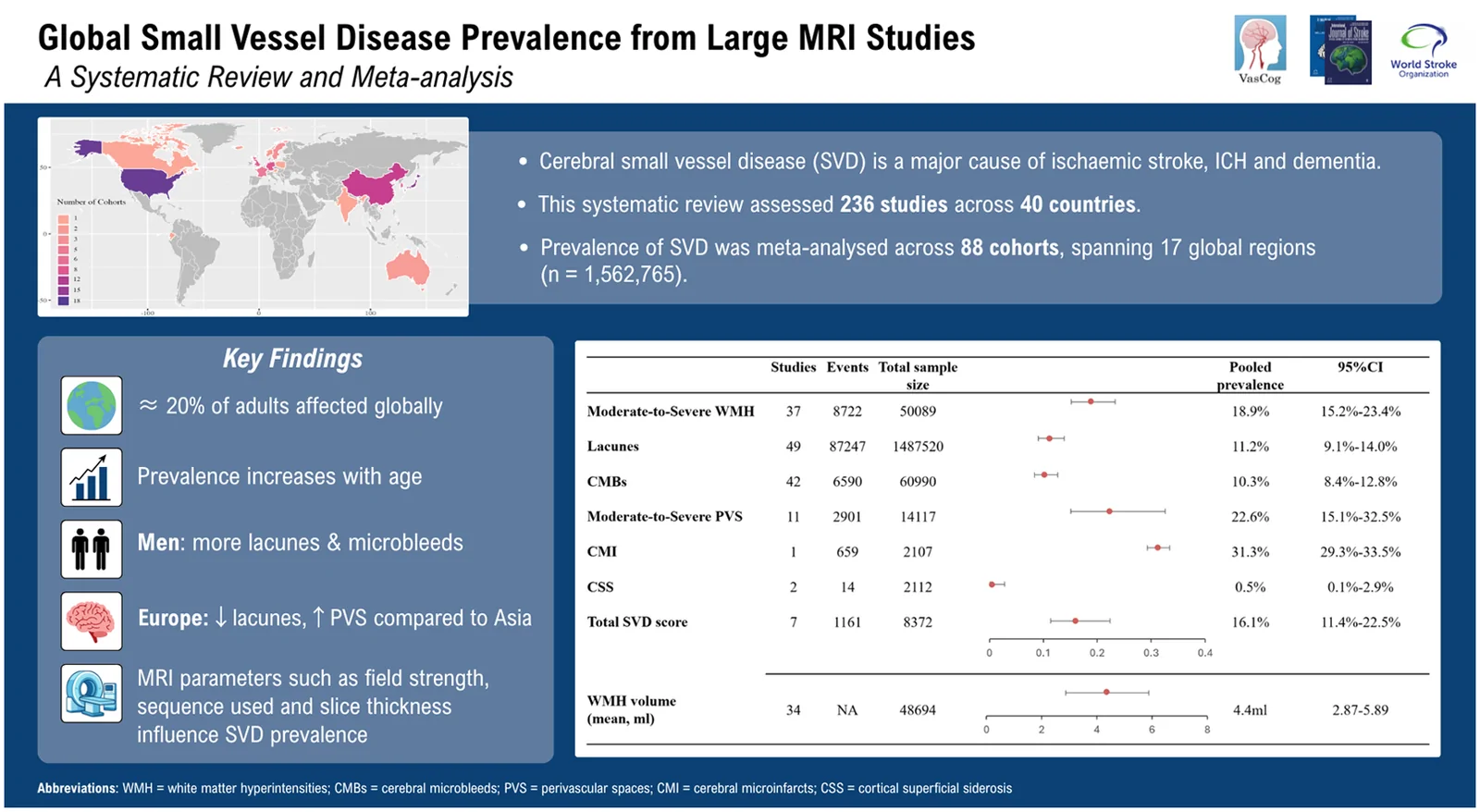

## Introduction

Cerebral small vessel disease (SVD) leads to about 25% of ischemic strokes and most hemorrhagic strokes in adults more than 65 years and is the major cause of vascular cognitive impairment. Other clinical symptoms include impaired mobility, gait, mood, and neurobehavioral disorders.^1^ The core SVD neuroimaging markers include recent small subcortical infarcts, lacunes (of presumed vascular origin), white matter hyperintensities (WMHs of presumed vascular origin), perivascular spaces (PVS), and cerebral microbleeds (CMBs).^2,3^ The challenge to report SVD prevalence arises as SVD is an umbrella term with multiple SVD neuroimaging manifestations. However, SVD neuroimaging markers assessed from a range of magnetic resonance imaging (MRI) sequences are still the most robust for estimating SVD prevalence. Furthermore, it is unclear how SVD prevalence may vary depending on the region (separated by geography or gross national income per capita), age, and sex. In addition, SVD prevalence may vary depending on the methodology, such as MRI machine field strength, sequence used, slice thickness, and analytical techniques to assess SVD.^3^ It is, therefore, important to understand how the MRI acquisition protocol may add to the variability of SVD prevalence on top of the differences observed in region, age, and sex. One previous review article reported the SVD prevalence of major population-based studies from high-income countries (HICs), showing the presence of WMH ranged 65–96%, the prevalence of lacunes as 8–31%, and of CMB, 3.1% to 15.3% (with mean age of subjects from various cohorts roughly ranging 53–76 years).^4^ Other studies may have reported the prevalence of SVD but have mainly focused on (1) only one (or a few) SVD marker(s), or (2) on single geographically defined cohorts, or (3) are mostly from HICs.

The global burden of SVD is likely to be enormous, but few report its prevalence according to world regions, mean age, and sex. An international workgroup composed of a panel of experts in the field of SVD, including the International Society of Vascular Behavioural and Cognitive Disorders (VasCog) members, was formed to initiate this study of global SVD prevalence. The workgroup first investigated the prevalence of SVD in low- and middle-income countries (LMICs).^5^ The current article focuses on the global SVD prevalence. Our previous systematic review and meta-analysis revealed the community-based prevalence of SVD (with a median age of 51.4) in LMICs as follows: the overall prevalence of moderate-to-severe WMH was 20.5%; of lacunes, 0.8%; of CMB, 10.7%; and of moderate-to-severe PVS, 25.0%.^5^ For more information on the age-stratified, regional SVD prevalence, as well as the SVD prevalence in dementia and stroke studies in LMICs, please refer to the previous publication.^5^

This systematic review and meta-analysis aimed to summarize evidence and provide estimates of the global prevalence of SVD. In addition, to determine whether factors such as (1) regions, (2) age groups, (3) sex, and (4) MRI acquisition parameters used, such as field strength, sequence, and slice thickness, would affect the reported SVD prevalence.

## Methods

An international workgroup composed of a panel of experts in the field of SVD, including the VasCog members, was formed to initiate this review and coordinated by the research group from the Division of Neurology, Department of Medicine and Therapeutics, Chinese University of Hong Kong (CUHK). The protocol was registered on PROSPERO (CRD42022311133).

### Search strategy and selection criteria

For this systematic review and meta-analysis, we first searched on PROSPERO to ensure there were no existing and ongoing reviews on a similar topic. Then, we searched Ovid MEDLINE and EMBASE databases from 1 January 2000 until 31 March 2024 for articles reporting the prevalence of SVD, without language restrictions (see Supplemental Material Section A). The Preferred Reporting in Systematic Review and Meta-Analysis (PRISMA) guidelines were used. We also conducted an additional hand search on Google Scholar to include relevant articles. The literature search consisted of three main concepts: “SVD,” “prevalence,” “global and population-based.” For SVD, we referred to the STandards for ReportIng Vascular changes on nEuroimaging (STRIVE) classifications of SVD^2,3^ and search strategies from relevant reviews.^5,6^ For more information on the search terms, please refer to Supplemental Material Section A. For “prevalence,” we used the terms “global burden of disease” or “prevalence” or “incidence” or “epidemiology,” including both MeSH or .mp. to increase search sensitivity. For global regions, we refer to the geographic region and Global Burden of Disease (GBD). GBD grouped the world into seven super-regions according to similar cause-of-death patterns.^7^ The seven super-regions are (1) high income (including Southern Latin America, Western Europe, North America, Australasia, and High-income Asia Pacific); (2) Latin America and Caribbean; (3) Sub-Saharan Africa; (4) North Africa and Middle East; (5) Southeast Asia, East Asia, and Oceania; (6) South Asia; and (7) Central Europe, Eastern Europe, and Central Asia. For population-based studies, we used “population-based,” “case–control study,” “cross-sectional study,” “twin study,” “prospective study,” “retrospective study,” or “cohort analysis.”

Inclusion criteria for the systematic review were published original research articles: (1) between 1 January 2000 to 31 March 2024, (2) without language restrictions, (3) assessed the prevalence of at least one type of SVD lesion on MRI, and (4) population-based study or community-based study with sample size ⩾400 subjects. Exclusion criteria included (1) SVD diagnosis without brain MRI (clinical only or with computed tomography (CT) only); (2) studies investigating genetic variants of SVD (e.g. Cerebral Autosomal Dominant Arteriopathy with Subcortical Infarcts and Leukoencephalopathy (CADASIL) and Cerebral Autosomal Recessive Arteriopathy with Subcortical Infarcts and Leukoencephalopathy (CARASIL)); (3) studies that included participants with specific neurological diseases; (4) studies that specifically recruited participants based on the presence of SVD neuroimaging markers; (5) non-human studies; and (6) case studies, letters, or studies not published in full (see Supplemental Material Section B).

### Study article selection

Title and abstract screening, full-text review, and data extraction were completed by at least two independent reviewers using the COVIDENCE software. For details of reviewers and their involvement in the review, please see Supplemental Material Section C.

Data extracted included authors’ last name, year of publication, journal name, study name, type of study, sample size, race, GBD, geographic region, age, proportion male (the number of males divided by the total number of subjects), years of education, and the criteria used to define the SVD marker.

For lacunes, CMB, CMI, and cortical superficial siderosis (CSS), the prevalence of any presence was extracted. For the visual rating of WMH grade, PVS grade, and total SVD score, the prevalence of subjects reaching moderate-to-severe levels was extracted according to study-specific cutoff values if available. Otherwise, moderate-to-severe levels were defined according to the standard criteria: score ⩾ 2 for moderate-to-severe WMH with reference to VasCog-2-WSO Criteria;^8,9^ >10 EPVS in basal ganglia or centrum semiovale for moderate-to-severe PVS; and the total SVD score ⩾ 2 points. The raw WMH volume, raw intracranial volume (ICV), and the ICV-corrected WMH volume or ratio were extracted if available. MRI acquisition details include the MRI manufacturer and model, MRI field strength, the sequence used for assessing each SVD marker, echo time, repetition time, inversion time, resolution, slice thickness, and matrix size. In addition, we extracted information specific to the selected SVD marker. For example, the method of visual rating and cutoff applied for moderate-to-severe SVD, and the quantification tool used for WMH volume.

For multiple publications from the same cohort, we applied a predefined strategy for data extraction. A representative study from each cohort based was selected based on the following: a greater number of SVD markers reported, preference for more recent publications, and a larger sample size. There may be scenarios where more than one publication from the same cohort was used to extract complementary information on SVD prevalence. For example, if one publication reported lacune prevalence in 1000 subjects, while another publication from the same cohort and recruitment phrase reported both lacune and CMB prevalence in 600 subjects, lacune prevalence was extracted from the study with a larger sample size (first publication), and CMB prevalence was extracted from the latter publication. For further information on the complete list for systematic review and meta-analysis, please refer to the Supplemental Tables 1 and 2, respectively. Disagreements were resolved by team discussion (B.Y.K.L., Y.C., and V.C.T.M.).

### Quality assessment

Study quality was assessed using the Risk of Bias for Non-randomized Studies tool based on the representativeness of the sample (definition of target population, sampling, participant selection, response rate, etc.), data collection (methods and criteria), results reporting, and statistical analysis.^10,11^ Risk-of-bias assessments were performed by at least two independent researchers. Disagreements were resolved by researchers in the team (B.Y.K.L. and Y.C.).

### Data synthesis and analyses

In the primary analyses, the overall pooled prevalence estimate of SVD biomarkers was calculated by using a random-effects model with an inverse variance weighting.^12^ The results were computed using logit transformation and presented after back transformation to natural proportions. We used Cochran’s Q and the I^2^ statistic to assess between-study heterogeneity (with a p < 0.05 indicating significant heterogeneity; I^2^ ≈ 25%, 50%, and 75% interpreted as low, moderate, and high, with ⩾75% substantial), and we prespecified random-effects models to account for expected variation. Publication bias was assessed using funnel plots and Egger’s regression test. Random-effects meta-regression was performed to examine whether prevalence estimates varied by GBD region, geographic region, mean age group, sex, and study design. For the analysis of GBD regions, since previous studies have reported that better socioeconomic status is associated with lower SVD burden,^13^ we dichotomized the studies into HICs, the super-regions classified as high income in GBD classification) and LMICs (all the other regions), using HICs as the reference. For the analysis of geographic region, we grouped and compared studies from each continent according to geographical location. As there were only two studies from Australia and one study from South America (Ecuador), we excluded those studies from the geographic region analysis. No eligible studies were available from Africa or Antarctica. Thus, the final analyses were conducted among Asia, North America, and Europe, with Asia used as the reference. For age-based analysis, studies were categorized by mean age (<60, 60–69, 70–79, and ⩾80 years), with <60 years as the reference. For the analysis of study design, we categorized the studies into two groups based on their recruitment methods, as described in the original methodology: population-based or community-based and health check-up studies. The population-based or community-based group was used as the reference to analyze the influence of recruitment method on the prevalence of SVD biomarkers. To investigate sex differences in SVD prevalence, we conducted random-effects meta-analyses. For binary outcomes (WMH, lacunes, PVS, and CMB), we used the Mantel-Haenszel method to calculate pooled risk differences (RDs).^14^ For continuous outcomes (WMH volume), we used inverse variance weighting to calculate mean differences (MDs).

To explore the association between MRI acquisition parameters and the pooled SVD prevalence, we also conducted random-effects meta-regression using each MRI acquisition parameter as an independent variable and each SVD marker as a dependent variable. Details of the MRI-related acquisition parameters were also extracted for each SVD marker (Supplemental Tables 3–5).

For all meta-regression analyses, we adjusted for potential confounders, including mean age, proportion male, and Human Development Index (HDI), except for the mean age group, which only adjusted for proportion male and HDI. HDI is a composite measure that summarizes average achievements in three dimensions: health (life expectancy at birth), education (mean years of schooling for adults aged ⩾25 and expected years of schooling for children of school-entering age), and standard of living (gross national income per capita, using the logarithm of income).^7^ HDI is computed as the geometric mean of the three-dimensional indices. For each study, we obtained the HDI value for each country (or territory) and the corresponding mean recruitment year. HDI values are available online (https://hdr.undp.org/data-center/human-development-index#/indicies/HDI).

All meta-analyses, meta-regression, and subgroup analyses were performed in R programming (Version 4.4.3) using “metafor” and “meta” packages. A two-tailed p value < 0.05 was considered significant for all analyses.

## Results

Our search identified 14,582 studies, of which 2604 were duplicates (Figure 1). Of the 11,978 articles screened, 563 were eligible for full-text screening. We performed an additional hands-on search and identified 28 additional publications. Then, 345 articles were excluded from the review as they did not fit the inclusion criteria (Figure 1 and Supplemental Material Section B). Of the remaining 246 studies, 161 were excluded as they were repeated publications from the same cohort (n = 156) or did not include standardized reporting of SVD prevalence (i.e. data were being log-transformed or reporting prevalence of a marker only in a subregion of the brain) (n = 5). Eighty-five studies (88 cohorts) spanning 17 global regions (n = 1,562,765) were included in the meta-analysis. For an overview of all included studies in the systematic review and meta-analysis, please refer to Supplemental Tables 1 and 2, respectively.

**Figure 1.**
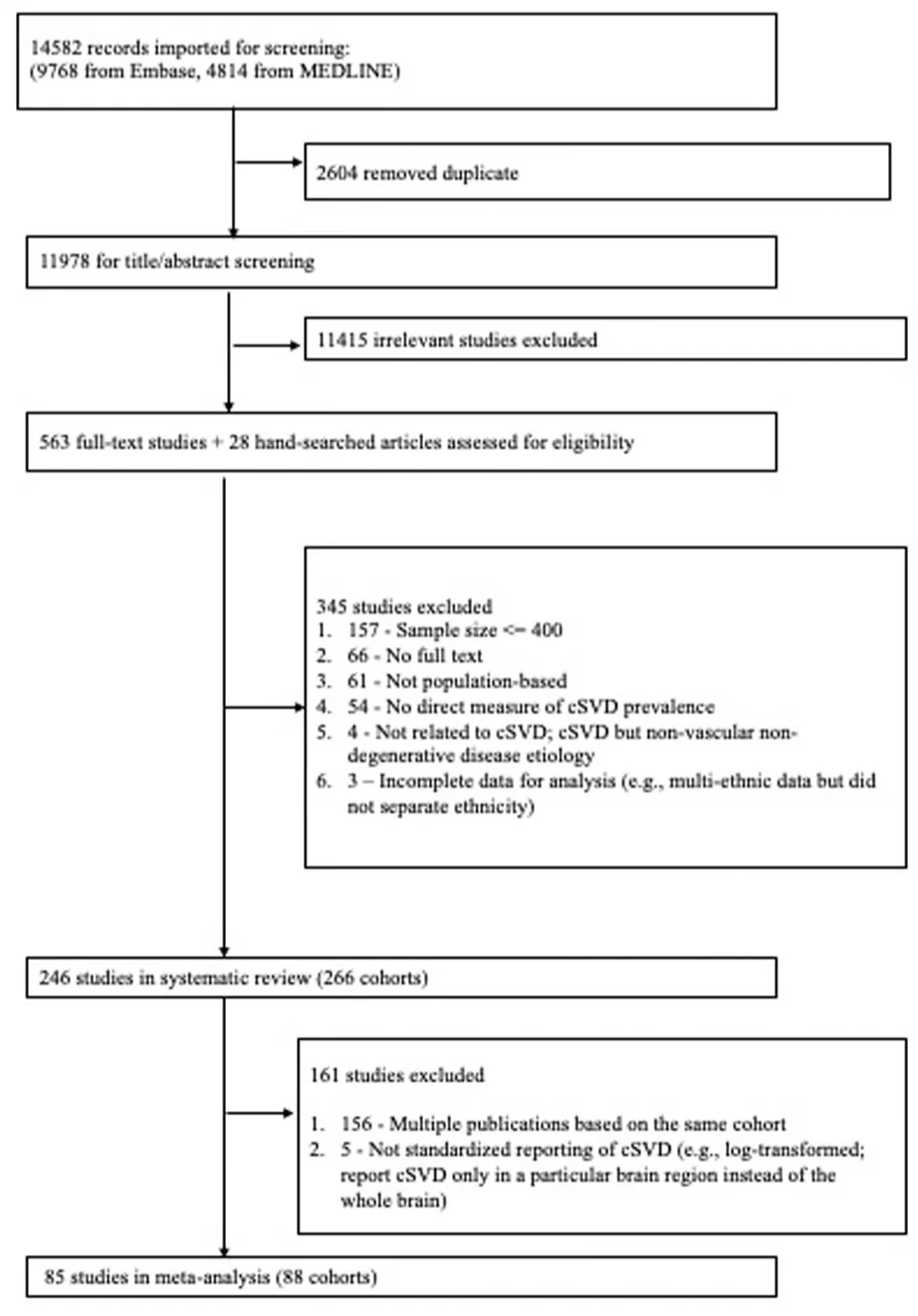
PRISMA flow diagram for systematic review and meta-analysis of studies to investigate the global prevalence of cerebral small vessel disease.

The mean age of all cohorts was 64.4 (ranged 46.4–81.6). The proportion of male subjects was 47.8% (ranged 33.8–53.6%) (see Table 1 and Supplemental Table 2). Most studies were from HICs (Table 1 and Figure 2), with the highest number of publications from the United States (n = 18), followed by Japan (n = 15). There was one publication each from Latin America and the Caribbean, South Asia, and Central Europe, Eastern Europe, and Central Asia that fitted the inclusion criteria. No population-based studies with a sample size larger than 400 were found in Sub-Saharan Africa, North Africa, and the Middle East. From our previous publication on LMICs, there are existing studies on the prevalence of SVD, but they focused only on the stroke population.^5^

**Table 1.** Characteristics of the studies included in the meta-analyses.

		High-income countries	Latin America and Caribbean	Southeast Asia, East Asia, and Oceania	South Asia	Central Europe, Eastern Europe, and Central Asia
Number of cohorts		73	1	12	1	1
Regions included		USA (n = 18), Japan (n = 15), Germany (n = 8), UK (n = 8), South Korea (n = 6), France (n = 5), Netherlands (n = 3), Sweden (n = 3), Australia (n = 2), Norway (n = 2), Austria (n = 1), Canada (n = 1), Iceland (n = 1)	Ecuador (n = 1)	China (n = 12)	India (n = 1)	Poland (n = 1)
Mean age group, years (N (%))	<60	25 (34.2%)	0 (0.0%)	7 (58.3%)	0 (0.0%)	0 (0.0%)
	60–69	23 (31.5%)	0 (0.0%)	3 (25%)	1 (100.0%)	1 (100.0%)
	70–79	23 (31.5%)	1 (100.0%)	2 (16.7%)	0 (0.0%)	0 (0.0%)
	80–89	2 (2.7%)	0 (0.0%)	0 (0.0%)	0 (0.0%)	0 (0.0%)
	⩾90^a^	0 (0.0%)	0 (0.0%)	0 (0.0%)	0 (0%)	0 (0%)
Male (%)		48.5%	43.40%	44.50%	53.60%	33.80%
Mean HDI (based on the mean period in which the MRI was performed)		0.9	0.8	0.7	0.6	0.8
Sample size (N (%))	401–1500	51 (69.9%)	1 (100.0%)	9 (75%)	1 (100.0%)	1 (100.0%)
	1501–3000	13 (17.8%)	0 (0.0%)	0 (0%)	0 (0.0%)	0 (0.0%)
	⩾ 3001	9 (12.3%)	0 (0.0%)	3 (25%)	0 (0.0%)	0 (0.0%)
Year of recruitment (N (%))	1988–1999	9 (12.3%)	0 (%)	0 (%)	0 (%)	0 (%)
	2000–2009	31 (42.5%)	0 (%)	1 (8.3%)	0 (%)	1 (100%)
	2010–2019	33 (45.2%)	1 (100%)	11 (91.7%)	1 (100%)	0 (%)
	>2019^b^	0 (%)	0 (%)	0 (%)	0 (%)	0 (%)
SVD lesions (N (%) or mean)	Moderate-to-severe WMH	18.3% (n = 26)	29.2% (n = 1)	20.1% (n = 8)	20.7% (n = 1)	13.4% (n = 1)
	WMH vol (mL)	4.6 (n = 29)	/	3.4 (n = 4)	/	1.9 (n = 1)
	Presence of lacune	11.1% (n = 38)	11.4% (n = 1)	12.7% (n = 9)	/	5.1% (n = 1)
	Presence of CMB	9.8% (n = 31)	16.3% (n = 1)	13.2% (n = 9)	/	4.0% (n = 1)
	Moderate-to-severe PVS	22.2% (n = 6)	31.0% (n = 1)	21.4% (n = 4)	/	/
	Presence of CMI	31.3% (n = 1)	/	/	/	/
	Presence of CSS	0.5% (n = 2)	/	/	/	/
	Total SVD score ⩾ 2)	14.8% (n = 3)	24.9% (n = 1)	15.9% (n = 3)	/	/
STRIVE criteria used (N (%))^c^		67 (91.8%)	1 (100.0%)	12 (100.0%)	1 (100.0%)	1 (100.0%)
MRI field strength ⩾ 3T (N (%))		20 (27.3%)	0 (0.0%)	10 (83.3%)	0 (0.0%)	0 (0.0%)
Risk-of-bias assessment (overall quality score) (mean score out of 8)		7.6	8	7.9	7	8

a Please note there are studies with subjects more than 90 years old, however, this figure refers to the SVD prevalence with respect to the average age of the cohort.

b Note that data were extracted from publications between the years 2000 to 2024; however, years of recruitment for all cohorts were until 2019.

c For studies published before the STRIVE 1 criteria (before 2013), we evaluated whether the description of each SVD marker was consistent with that as defined in the STRIVE criteria. If so, the study was considered to have used the STRIVE criteria.

**Figure 2.**
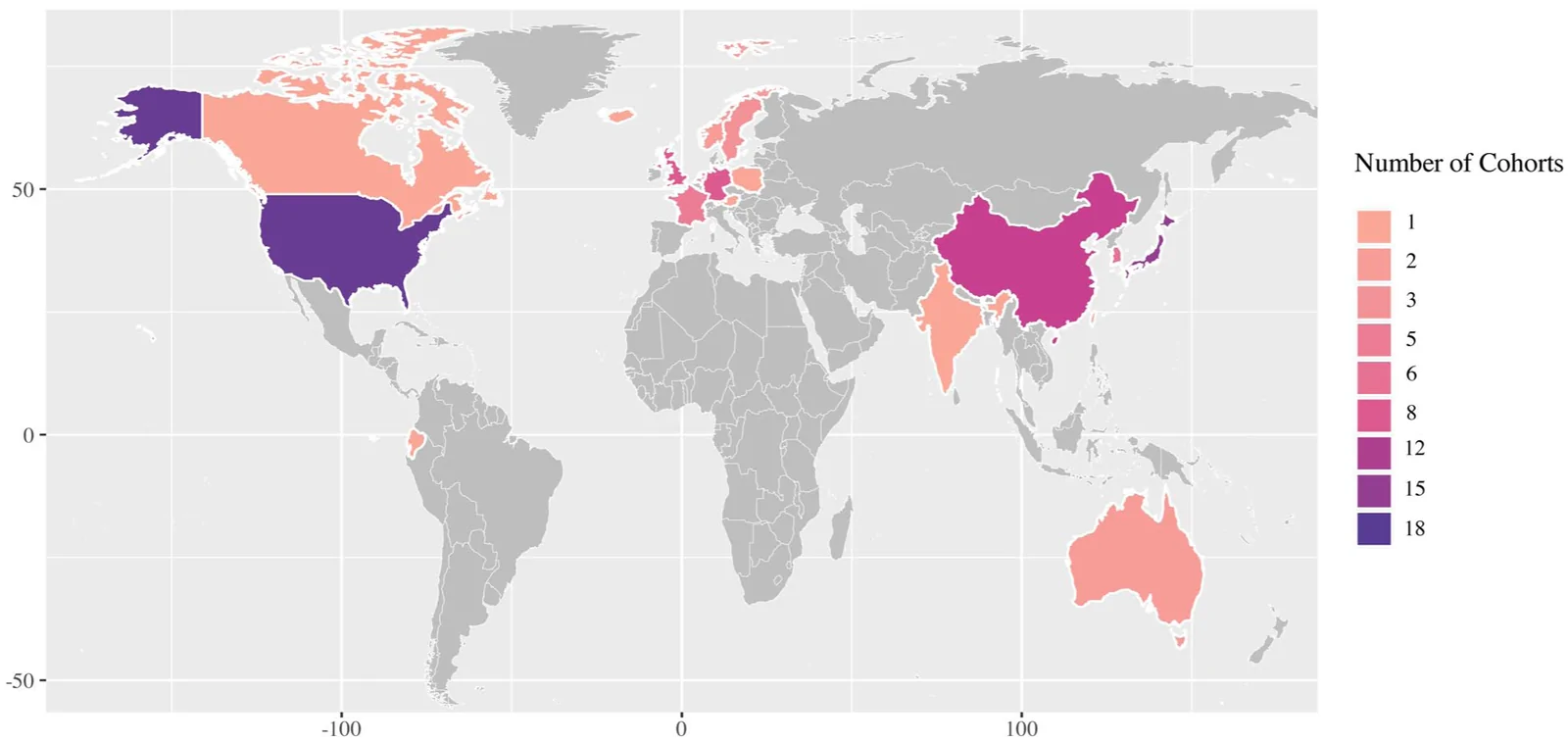
The global distribution of studies investigating cerebral small vessel disease prevalence included in the systematic review and meta-analysis.

Most cohorts recruited subjects after 2010, especially for Southeast Asia, East Asia, and Oceania. Sample sizes were typically under 1500 participants. Thirteen studies exceeded 3000 subjects (nine from HICs and three from Southeast Asia, East Asia, and Oceania), with the largest Chinese study including 1,431,527 subjects.^15^ Most publications used the STRIVE criteria when reporting (82 out of 88 cohorts; ⩾91.8%). Publications from Southeast Asia, East Asia, and Oceania used MRI magnetic field strength of 3T or higher (83.3%), while only 27.3% used 3T or higher MRI in HICs (see Table 1).

## Global prevalence of SVD markers and their associations with regions, mean age and sex

### Global prevalence of WMH, lacunes, CMB, and PVS

A total of 37 studies (mean age ± SD: 63.1 ± 7.4, 50,089 participants) reported the prevalence of moderate-to-severe WMH by visual rating, with a global pooled prevalence of 18.9% (95% confidence interval (CI): 15.2–23.4%). In addition, 34 studies including 48,694 participants (mean age ± SD: 64.8 ± 8.3) reported automatically segmented WMH volume, yielding a global pooled mean of 4.4 mL (95% CI: 2.9–5.9 mL). The prevalence of lacunes was examined in 49 studies encompassing 1,487,520 participants (mean age ± SD: 65.3 ± 8.0), yielding a global pooled prevalence of 11.2% (95% CI: 9.1–14.0%). For CMB, data were available from 42 studies with 60,990 participants (mean age ± SD: 65.5 ± 8.0), yielding a pooled global prevalence of 10.3% (95% CI: 8.4–12.8%). Moderate-to-severe PVS were reported in 11 studies with 14,117 participants (mean age ± SD: 63.9 ± 8.3), with a global pooled prevalence of 22.6% (95% CI: 15.1–32.5%) (Figure 3). For forest plots of each SVD marker, please refer to Supplemental Figures 1–5.

**Figure 3.**
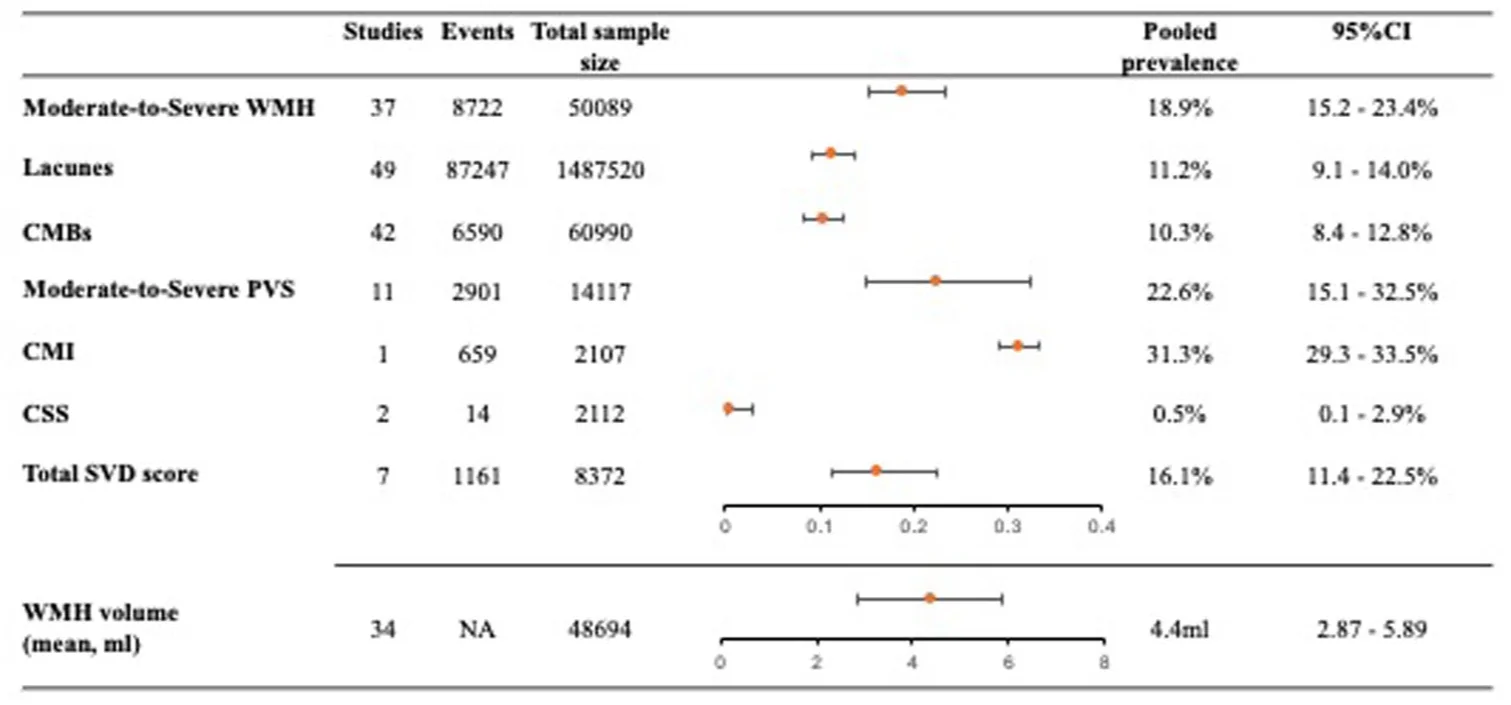
The pooled prevalence of all cerebral small vessel disease markers. Orange points represent pooled prevalence estimates with horizontal lines indicating 95% confidence intervals. The left columns show the number of contributing studies, the number of events (participants with the condition), and the total sample size for each marker. Prevalence estimates are presented as percentages with 95% confidence intervals. The analysis included moderate-to-severe WMH, lacunes, cerebral microbleeds (CMBs), moderate-to-severe perivascular spaces (PVS), cortical microinfarcts (CMIs), CSS, and total SVD score. WMH volume is presented separately as a continuous measure (mean volume in mL).

### Other SVD markers

Only one population-based study assessed cortical microinfarcts (mean age: 75.7, 2107 participants), and the prevalence was 31.3%. Two studies reported the prevalence of CSS (mean age ± SD: 70.9 ± 2.2, 2112 participants), and the pooled prevalence was 0.5%. A few studies assessed SVD using the total SVD score with a pooled prevalence of 16.1% (mean age ± SD: 63.8 ± 7.7, 8372 participants) (Figure 3). In high-income regions (n = 3), 14.8% had a total SVD score ⩾2; 15.9% in Southeast Asia, East Asia, and Oceania (n = 3) and 24.9% in Latin America and Caribbean (n = 1) (Supplemental Figures 6–8) Due to the relatively small sample size in these markers, no subgroup analysis was performed.

### The association between the prevalence of SVD markers and world regions

We examined the association between SVD markers across different regional classifications stratified by GBD economic regions and geographical regions, and all meta-regressions were adjusted for mean age, proportion male, and HDI. When world regions were stratified by economic status, no statistically significant difference was observed across any SVD markers between LMICs and HICs (Table 2). When comparing geographic regions, Europe demonstrated a lower prevalence of lacunes (7.3% vs 13.2%; adjusted odds ratio (aOR) [95% CI]: 0.45 [0.30–0.68], p < 0.001) but a higher prevalence of PVS (30.9% vs 19.6%; aOR [95% CI]: 12.15 [2.12–69.46], p = 0.005) compared with Asia. No significant differences were found for moderate-to-severe WMH, WMH volume, or CMB between the geographical regions assessed. The prevalence in North America showed no statistically significant differences compared with Asia across all SVD markers (Table 2).

**Table 2.** Meta-regression analysis of the prevalence of cerebral small vessel disease by regions, age group, and study design.

	Moderate-to-severe WMH				WMH volume				Lacunes				CMB				Moderate-to-severe PVS			
	N	Pooled prevalence (95% CI)	Adjusted OR (95% CI)	Adjusted p value	N	Pooled prevalence (mL, 95% CI)	Adjusted MD (95% CI)	Adjusted p value	N	Pooled prevalence (95% CI)	Adjusted OR (95% CI)	Adjusted p value	N	Pooled prevalence (95% CI)	Adjusted OR (95% CI)	Adjusted p value	N	Pooled prevalence (95% CI)	Adjusted OR (95% CI)	Adjusted p value
World regions (by economic status)																				
High-income countries	26	18.3% (13.5–24.4)	Ref	Ref	29	4.6% (2.9–6.3)	Ref	Ref	38	11.1% (8.6–14.2)	Ref	Ref	31	9.8% (7.5–12.7)	Ref	Ref	6	22.2% (11.8–37.9)	Ref	Ref
Low- and middle-income countries	11	20.2% (17.3–23.4)	0.83 (0.30–2.32)	0.729	5	3.1% (0.2–6.0)	3.24 (−3.94 to 10.42)	0.376	11	11.6% (8.1–16.5)	0.68 (0.30–1.55)	0.358	11	11.8% (9.0–15.3)	1.40 (0.56–3.49)	0.474	5	23.1% (12.9–37.8)	0.49 (0.03–7.82)	0.613
World regions (by geography)																				
Asia	24	18.2% (14.1–23.1)	Ref	Ref	5	3.6% (0.7–64)	Ref	Ref	22	13.2% (10.4–16.5)	Ref	Ref	22	8.8% (6.8–11.3)	Ref	Ref	5	19.6% (10.7–33.1)	Ref	Ref
Europe	8	20.9% (11.9–33.9)	1.30 (0.66–2.54)	0.443	24	3.9% (2.2–5.6)	−0.74 (−5.97 to 4.49)	0.782	15	7.3% (4.6–11.4)	0.45 (0.30–0.68)	<0.001	11	9.7% (5.8–15.7)	1.05 (0.62–1.78)	0.858	4	30.9% (17.0–49.5)	12.15 (2.12–69.46)	0.005
North America	4	17.0% (7.0–35.8)	0.73 (0.30–1.75)	0.477	4	5.5% (0.4–10.6)	1.02 (−5.57 to 7.60)	0.762	9	15.9% (9.4–25.5)	0.72 (0.44–1.18)	0.197	7	16.2% (10.9–23.4)	1.23 (0.62–2.42)	0.555	1	7.9% (7.9–7.9)	2.00 (0.27–14.97)	0.499
Age range^a^																				
<60	15	16.1% (10.8–23.3)	Ref	Ref	12	2.5% (0.0–5.0)	Ref	Ref	16	6.7% (4.6–9.8)	Ref	Ref	14	7.1% (4.8–10.3)	Ref	Ref	5	27.2% (15.2–44.0)	Ref	Ref
60–69	15	16.8% (12.5–22.1)	1.04 (0.60–1.81)	0.879	9	2.8% (1.9–3.8)	0.26 (−3.39 to 3.91)	0.890	16	11.7% (9.2–14.9)	1.95 (1.20–3.15)	0.007	13	10.0% (6.9–14.3)	1.60 (0.93–2.75)	0.089	2	8.9% (7–11.0)	0.27 (0.07–1.01)	0.052
70–79	6	33.3% (27.0–40.3)	2.58 (1.22–5.46)	0.013	13	7.2% (4.6–9.8)	4.65 (1.41–7.89)	0.005	16	15.9% (11.7–21.2)	2.94 (1.79–4.85)	<0.001	14	14.7% (11.4–18.8)	2.43 (1.39–4.23)	0.002	4	27.2% (16.5–41.3)	1.08 (0.36–3.27)	0.895
80–89	1	27.1% (27.1–27.1)	1.88 (0.30–11.71)	0.498	NA	NA	NA	NA	1	43.6% (43.6–43.6)	9.53 (1.68–54.16)	0.011	1	14.0% (14.0–14.0)	2.42 (0.57–10.24)	0.229	NA	NA	NA	NA
Study design																				
Population-based or community-based	29	21.1% (17.2–25.6)	Ref	Ref	34	4.4% (2.9–5.9)	Ref	Ref	41	11.7% (9.2–14.9)	Ref	Ref	33	11.9% (9.5–14.9)	Ref	Ref	9	23.2% (14.3–35.4)	Ref	Ref
Health check-up	8	12.3% (6.4–22.2)	0.67 (0.36–1.27)	0.221	0	NA	NA	NA	8	8.9% (6.6–11.7)	1.50 (0.86–2.61)	0.151	9	5.8% (4.6–7.4)	0.78 (0.47–1.31)	0.350	2	20.0% (9.0–38.7)	0.37 (0.03–4.99)	0.451

a Denotes analyses were performed with male proportion and HDI as covariates, while other analyses were performed with age, male proportion, and HDI as covariates.

### The association between the prevalence of SVD markers and age

We examined the association between SVD markers across different age groups, using studies with a mean age <60 years as the reference group and adjusted for proportion male and HDI. Significant age-related increases were observed for multiple SVD markers (Table 2). For moderate-to-severe WMH by visual rating, the 70–79 age group showed significantly higher prevalence compared to those aged <60 years (33.3% vs 16.1%; aOR [95% CI]: 2.58 [1.22–5.46], p = 0.013), while the 60–69 and 80–89 age groups demonstrated no significant differences. WMH volume showed an increase in volume observed in the 70–79 age group, but with marginal significance (7.2 mL vs 2.5 mL, p = 0.005). Lacunes demonstrated an age-related increase in prevalence, with significant elevations beginning in the 60–69 age group (11.7%; aOR [95% CI]: 1.95 [1.20–3.15], p = 0.007) compared to 6.7% in those <60 years. Lacune prevalence further increased in the 70–79 age group (15.9%; aOR [95% CI]: 2.94 [1.79–4.85], p < 0.001) and reached the highest levels in the 80–89 age group (43.6%; aOR [95% CI]: 9.53 [1.68–54.16], p = 0.011). CMB showed significant increase in prevalence only in the 70–79 age group (14.7% vs 7.1%; aOR [95% CI]: 2.43 [1.39–4.23], p = 0.002). Moderate-to-severe PVS demonstrated no significant age-related differences across all age groups (all p > 0.05).

### The association between the prevalence of SVD markers and study design

When analyzing the influence of study design on SVD marker prevalence, population-based or community-based studies served as the reference against studies with subjects recruited from a health check-up. Health check-up studies consistently demonstrated lower prevalence estimates across most SVD markers, with notable differences observed for cerebral microbleeds (CMBs) (11.9% vs 5.8%, aOR: 0.78, 95% CI: 0.47–1.31, p = 0.350), WMH visual rating (21.1% vs 12.3%, aOR: 0.67, 95% CI: 0.36–1.27, p = 0.221), and lacunes (11.7% vs 8.9%, aOR: 1.50, 95% CI: 0.86–2.61, p = 0.151). After adjusting for mean age, proportion male, and HDI, all comparisons did not reach statistical significance.

### Sex-stratified SVD prevalence

Only 20 out of 88 cohorts reported the prevalence stratified by sex. Meta-regression analyses were performed to explore the association between sex and SVD prevalence (see Supplemental Figure 9). As there are a limited number of studies, results are described only if there were five or more studies for a particular SVD marker, which includes moderate-to-severe WMH, lacune, and CMB counts. Overall, males demonstrated significantly higher prevalence of lacunes (RD [95% CI]: 0.05 [0.01; 0.10]) and CMB (RD [95% CI]: 0.05 [0.01; 0.09]) compared with females. WMH volume showed a trend toward higher values in males but did not reach statistical significance (MD [95% CI]: 1.62 [−0.82; 4.06]). There were fewer than five studies on the sex-stratified prevalence of moderate-to-severe WMH and moderate-to-severe PVS, and the limited number of studies prevented us from drawing further conclusions.

## MRI parameters contributing to SVD prevalence

The results of the meta-regression analyses examining the impact of MRI acquisition parameters on SVD prevalence are shown in Figure 4. All analyses were adjusted for mean age, proportion male, and HDI. For details of the MRI-related acquisition parameters of the studies, please refer to Supplemental Tables 3–5.

**Figure 4.**
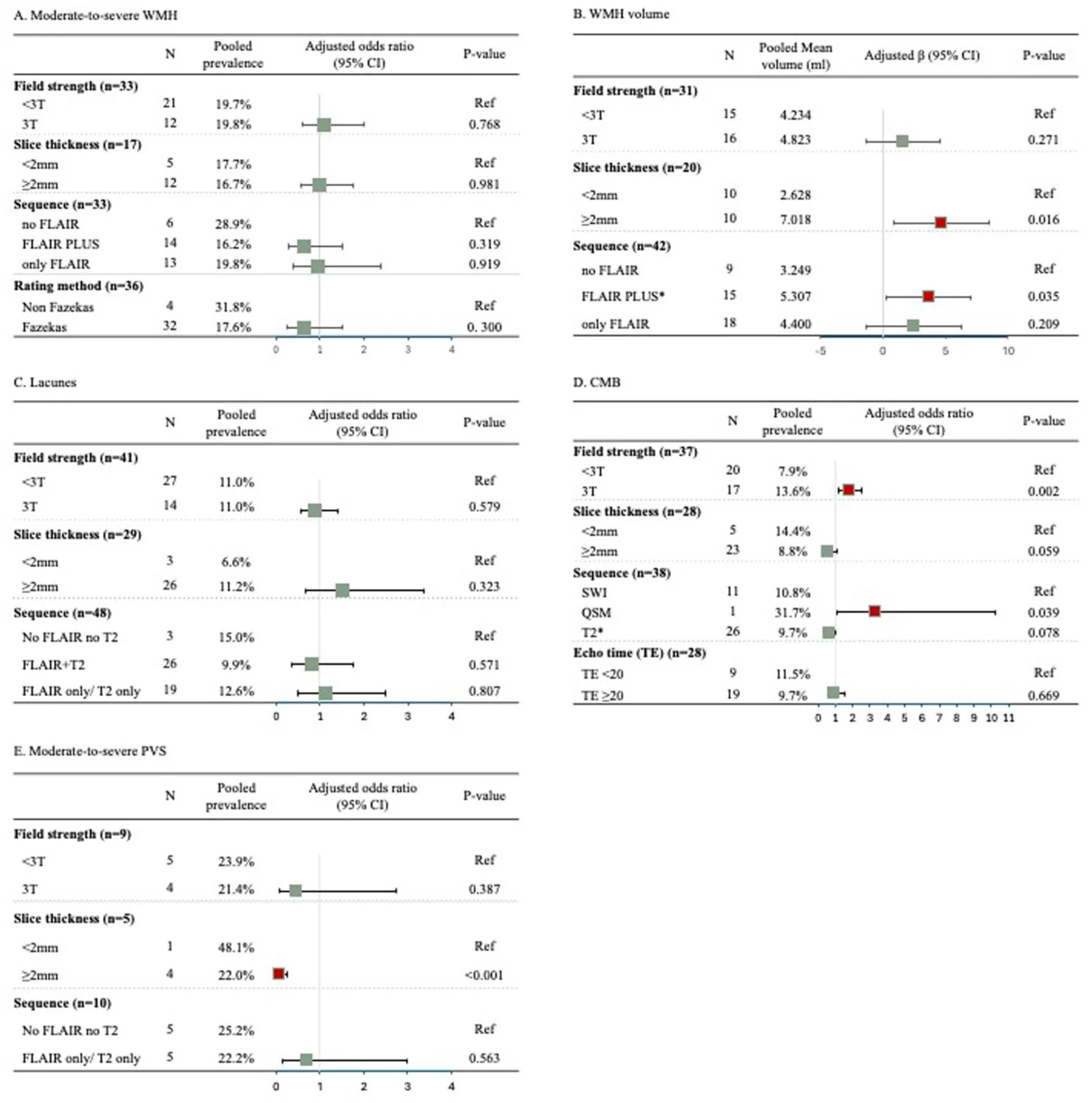
The association between MRI acquisition parameters and small vessel disease markers. Forest plots displaying adjusted associations between MRI acquisition parameters and five small vessel disease markers: (a) moderate-to-severe white matter hyperintensities (WMH), (b) WMH volume, (c) lacunes, (d) cerebral microbleeds (CMB), and (e) moderate-to-severe perivascular spaces (PVS). All analyses were adjusted for mean age, proportion male, and Human Development Index (HDI). For binary outcomes (a, c, d, e), data are presented as aORs with 95% confidence intervals. For the continuous outcome (b), data are presented as adjusted β coefficients (mL) with 95% confidence intervals. Square markers represent point estimates, with horizontal lines indicating 95% confidence intervals. Red markers indicate statistically significant associations (p < 0.05), while green markers indicate non-significant associations. Reference groups are marked as “Ref” and positioned at the null effect line (log OR = 0 for binary outcomes). Numbers in the left columns indicate the number of studies contributing to each comparison. FLAIR PLUS* indicate studies using FLAIR sequences combined with T1- or T2-weighted imaging.

For moderate-to-severe WMH by visual rating, no significant association between prevalence and MRI acquisition parameters was observed. For WMH volume measurements, slice thickness and sequence selection significantly influenced the pooled estimates. FLAIR slice thickness ⩾2 mm was associated with larger WMH volume estimates compared with thinner slices (<2 mm) (7.0 mL vs 2.6 mL; aβ [95% CI]: 4.69 [0.87–8.51], p = 0.016). Studies using FLAIR sequences combined with T1- or T2-weighted imaging yielded substantially higher mean WMH volumes compared to those without using the FLAIR for assessment (5.3 vs 3.2 mL; adjusted β [95% CI]: 3.66 [0.25–7.06], p = 0.035). Raw WMH volumes were used in the primary meta-analysis to maximize sample size, even though some studies report normalized volumes or log-transformed values. Supplemental analyses of studies providing both raw and normalized volumes (n = 5) showed similar results with no significant differences in heterogeneity (Supplemental Figure 10). Lacune prevalence showed minimal sensitivity to MRI acquisition parameters. Neither field strength nor slice thickness variations significantly contributed to prevalence differences across studies.

CMB prevalence, however, was substantially influenced by MRI acquisition parameters. Higher field strength (3T) significantly increased CMB detection rates compared to <3T (13.6% vs 7.9%; aOR [95% CI]: 1.77 [1.22–2.57], p = 0.002). MRI slice thickness is marginally significant in its association with CMB count, with thicker acquisitions (>2 mm) demonstrating lower reported prevalence than thinner slice (<2 mm) (8.8% vs 14.4%; aOR [95% CI]: 0.59 [0.34–1.02], p = 0.059). Sequence selection significantly influenced CMB detection. Studies using QSM showed substantially higher odds compared with SWI (31.7% vs 10.8%; aOR [95% CI]: 3.30 [1.06–10.21], p = 0.039), although this finding should be interpreted cautiously given only one study employed QSM. The comparison between T2*(GRE) and SWI sequences showed a trend toward lower CMB odds with T2* (9.7% vs 10.8%; aOR [95% CI]: 0.68 [0.44–1.04], p = 0.078). Echo time variations did not significantly affect detection rates (TE < 20 ms vs TE ⩾ 20 ms: aOR [95% CI]: 0.89 [0.53–1.50], p = 0.669). PVS prevalence showed limited association with MRI acquisition parameters, although the small number of included studies (n = 11) constrains the robustness of these analyses. Slice thickness demonstrated a significant effect, with thicker acquisitions (>2 mm) showing substantially lower detection rates compared with thinner slices (<2 mm) (22.0% vs 48.1%; aOR [95% CI]: 0.09 [0.03–0.26], p < 0.001). However, this comparison is limited to only one study using <2 mm. Results with this limited number of studies showed that field strength and sequence selection had no significant effect on the prevalence of PVS.

### Heterogeneity, study quality, and risk-of-bias assessments

High heterogeneity was observed in the studies, and subgroup analyses were performed separating regions, mean age groups, and study design; however, the high heterogeneity remained (I² > 90%) (see Supplemental Figures 1–8, Supplemental Table 6).

Overall, the quality of cohorts included in this systematic review and meta-analysis was high (mean score 7.67 out of 8). The funnel plot showed good symmetry for moderate-to-severe WMH, lacunes count, and moderate-to-severe PVS. The regression test for funnel plot asymmetry of these markers showed there is no publication bias, except for WMH volume and CMB count (Supplemental Figure 11).

## Discussion

This is the first systematic review and meta-analysis to investigate the global prevalence of SVD. Of 14,582 studies identified, 246 studies spanning 40 global regions were included in the systematic review. In the meta-analysis, 85 studies spanning 17 global regions (n = 1,562,765) were included. The pooled prevalence of moderate-to-severe WMH was 18.9%, and the pooled mean of WMH volume was 4.4 mL. The pooled prevalences of lacunes, CMB, and moderate-to-severe PVS were 11.2%, 10.3%, and 22.6%, respectively.

### SVD prevalences across different regions

Our findings revealed that geographic location, rather than economic status, is associated with regional variations in SVD marker prevalence. No significant disparities emerged between LMICs and HICs across any SVD markers after adjusting for demographic and developmental factors. This finding challenges the assumption that economic status directly translates to differential SVD burden. More notably, the geographic comparison between Europe and Asia unveiled a paradoxical pattern: European populations exhibited significantly lower lacune prevalence (7.3% vs 13.2%), yet higher PVS burden (30.9% vs 19.6%) compared with Asian cohorts. This elevated rate of lacunes in Asia is consistent with reported higher rates of hypertension and diabetes,^16,17^ which are both independent risk factors for lacunes.^18^ There are limited data on the associations of PVS and ethnicity. In the Multi-Ethnic Study of Atherosclerosis, Chinese Americans showed significantly smaller temporal and frontoparietal region PVS volumes compared with White participants.^19^ The absence of WMH and CMB differences suggests these markers might represent more of a universal aging phenomenon. The normative age- and sex-specific WMH values from 15 population-based cohorts with wide geographical and ethnic coverage supported this finding by showing WMH volumes were comparable across different ethnic groups in various global regions, although non-White subjects were underrepresented.^20^ More studies regarding ethnic differences in SVD prevalence and its underlying mechanisms are warranted in the future.

### Increasing age with increasing SVD prevalence

Meta-regression showed that increasing age was a significant predictor of a higher prevalence of moderate-to-severe WMH, greater WMH volume, increasing numbers of lacunes, and CMBs when compared to the reference group (mean age <60 years). The prevalence of most SVD markers at a mean age range of 70–79 years was significantly higher than that of <60 years. There was no significant difference in PVS, although this may be due to the small number of studies (n = 6). Results from the 15 population-based studies further support this finding by showing both the WMH volume and its variance increased exponentially with age. For WMH volume, the median volume doubled approximately every 10 years of age increase.^20^ In addition to WMH, results from this meta-analysis highlighted that other SVD markers, such as the prevalence of lacune and CMB, also increase with age.

### Sex difference in SVD prevalence

Only 20 out of 88 studies had SVD prevalence reported separately for males and females, which limited the statistical power to explore sex differences. Overall, males demonstrated significantly higher prevalence of lacunes and CMB compared with females. WMH volume showed a trend toward higher values in males but did not reach statistical significance. The number of studies with sex-stratified SVD prevalence in moderate-to-severe WMH prevalence or moderate-to-severe PVS prevalence was limited (<5), which prevented us from drawing further conclusions. Males showing more moderate-to-severe SVD were described previously, but mainly in populations with clinical SVD rather than in population studies.^21^ It has previously been suggested that sex differences in SVD prevalence may not always be in the same direction, as SVD is not a single entity, and each SVD marker may reflect different pathophysiology. A large multi-cohort study with previous stroke or transient ischemic attack (38 cohorts from 17 countries) reported females showed more moderate-to-severe WMH,^22^ while males showed more lacunes and microbleeds.^22^ These patterns of sex differences in SVD prevalence were also observed in a large population-based study, in which the normative data of WMH showed females had a higher WMH volume, with a difference in age effect of 2 to 5 years in females compared with males.^20^ Similarly, for CMB, being male was associated with greater lobar CMB (but not for infratentorial or deep CMB) in a UK Biobank study.^23^ Our results for lacunes and CMB were consistent with previous studies showing male prevalence is higher.^22,23^ Sex-stratified prevalence should be reported in studies to better understand whether the differences observed are due to biological sex differences, recruitment bias, other lifestyle or genetic factors, and the sex-stratified results have clinical implications, such as personalised care.^21^

### MRI acquisition parameters and SVD prevalence

In this review, we assessed the impact of MRI acquisition parameters on the prevalence of SVD. Meta-regression analyses showed that MRI magnetic field strength, the MRI sequence used, and sequence slice thickness were significant contributors to the variability in the prevalence of SVD. Mean WMH volume and the prevalence of CMB are SVD markers most prone to the variability of MRI acquisition parameters. Our findings emphasised the importance of adopting a harmonised SVD MRI scanning protocol. For more information on how the MRI parameters may influence SVD prevalence, please refer to Supplemental Section D.

### Heterogeneity

In this systematic review and meta-analysis, we showed that the heterogeneities in all SVD markers were high, and the factors such as regions, age groups, study design, and MRI acquisition parameters did not significantly reduce the variance observed (see Supplemental Table 6). One reason for the high heterogeneity could be due to the variability naturally exhibited in the general ageing population. This study intended to investigate population-based and large community-based studies that reflect the true prevalence of SVD in the general population. Therefore, we did not exclude subjects with one or multiple vascular risk factors, a history of stroke or dementia, which will contribute to the heterogeneity of SVD prevalence observed. Note, however, that even though studies from health check-ups tend to have a lower SVD prevalence compared with other population- or community-based studies, there were no significant differences after adjustment for mean age, proportion male, and HDI. We speculate that factor(s) contributing to the heterogeneity of SVD prevalence, such as the presence of vascular risk factors, genetics, and other environmental factors that are not captured in this review, could contribute to the heterogeneity of SVD prevalence.^24^

Overall, there is a low bias in the studies as illustrated by the funnel plots, except for WMH volume and CMB counts. However, given the substantial heterogeneity in our meta-analyses, we interpret these correction results with considerable caution, as the funnel plot asymmetry may be due to heterogeneity rather than publication bias (6). There was also no major concern over the quality of studies (scores ranging from 7 to 8 out of 8).

### Limitations

First, our inclusion criteria include only studies with MRI and a sample size of 400 or above; the high research cost may limit the number of studies available from LMICs. However, MRI neuroimaging markers remain most robust to fully capture SVD prevalence; therefore, this criterion is essential to retain. We attempted to strike a balance in this aspect, keeping a moderate sample size enough to reflect population prevalence, but at the same time, capturing studies from most global regions. Second, while differences in SVD prevalence across regions were identified, data on ethnicity and SVD prevalence are limited and would be required to further understand the underlying mechanisms behind these observed differences. Third, in this study, we cannot disentangle whether the SVD prevalence increase is due to older age or survivorship bias.^25^ Fourth, this review concurs with previous studies that there are sex differences in SVD prevalence, such that there are higher prevalences of lacunes and CMBs in males. Future studies should consider correcting for other factors that may play an important role, such as severity and duration of cerebrovascular risk factors control, dietary factors, and genetics, to further understand the sex differences observed. Fifth, for certain biomarkers (e.g. WMH volume and CMB), we detected signals of potential publication bias through funnel plot asymmetry and Egger’s tests; however, the substantial heterogeneity in our analyses limits the reliability of standard correction methods such as trim-and-fill,^26^ as heterogeneity and publication bias can mutually confound each other, making it difficult to distinguish true missing studies from methodological and clinical variations between included studies.

### Summary

In summary, we assessed the global prevalence of SVD and showed that the pooled prevalence of moderate-to-severe WMH was 18.9%, and the pooled mean of WMH volume was 4.7 mL. The pooled prevalence of lacunes, CMB, and moderate-to-severe PVS was 11.2%, 10.3%, and 22.6%, respectively.

This meta-analysis documented regional, mean age, and sex differences in SVD prevalence. A lower lacune prevalence but higher PVS prevalence was found in Europe compared with Asia. Older mean age was the largest contributing factor to the global SVD prevalence. Males tend to have more lacunes and CMBs compared with females. Adopting a harmonised SVD MRI scanning protocol would allow optimal assessment of SVD prevalence globally and benefit SVD research. We also acknowledged a lack of studies from LMICs. More population-based studies are particularly needed from Sub-Saharan Africa, North Africa, and Middle East, as well as Latin America and Caribbean, South Asia, and Central Europe, Eastern Europe, and Central Asia to provide a truly global estimate of the SVD burden.

## Supplemental Material

sj-docx-1-wso-10.1177_17474930261441916 – Supplemental material for Global burden of cerebral small vessel disease determined from large MRI studies: A systematic review and meta-analysis Supplemental material, sj-docx-1-wso-10.1177_17474930261441916 for Global burden of cerebral small vessel disease determined from large MRI studies: A systematic review and meta-analysis by Bonnie Yin Ka Lam, Yuan Cai, Huijing Zheng, Jize Wei, Rufus Akinyemi, Geert Jan Biessels, Hilde van den Brink, Christopher Chen, Marco Duering, Deborah Gustafson, Saima Hilal, Vincent Ming Ho Hui, Rajesh Kalaria, SangYun Kim, Maggie Li Man Lam, Frank-Erik de Leeuw, Ami Sin Man Li, Hugh S Markus, Anna Marseglia, John O'Brien, Leonardo Pantoni, Perminder S Sachdev, Eric E Smith, Joanna M Wardlaw, Adrian Wong, Ho Ko and Vincent Chung Tong Mok in International Journal of Stroke

sj-xlsx-2-wso-10.1177_17474930261441916 – Supplemental material for Global burden of cerebral small vessel disease determined from large MRI studies: A systematic review and meta-analysis Supplemental material, sj-xlsx-2-wso-10.1177_17474930261441916 for Global burden of cerebral small vessel disease determined from large MRI studies: A systematic review and meta-analysis by Bonnie Yin Ka Lam, Yuan Cai, Huijing Zheng, Jize Wei, Rufus Akinyemi, Geert Jan Biessels, Hilde van den Brink, Christopher Chen, Marco Duering, Deborah Gustafson, Saima Hilal, Vincent Ming Ho Hui, Rajesh Kalaria, SangYun Kim, Maggie Li Man Lam, Frank-Erik de Leeuw, Ami Sin Man Li, Hugh S Markus, Anna Marseglia, John O'Brien, Leonardo Pantoni, Perminder S Sachdev, Eric E Smith, Joanna M Wardlaw, Adrian Wong, Ho Ko and Vincent Chung Tong Mok in International Journal of Stroke
