# Supplementary material for: Global burden of cerebral small vessel disease determined from large MRI studies: A systematic review and meta-analysis
Source: Int J Stroke. 2026 Apr 4;21(9):1284–98. doi: 10.1177/17474930261441916 (PMC13672879; doi:10.1177/17474930261441916)
Supplement: Supplementary file 1 [file sj-docx-1-wso-10.1177_17474930261441916.docx]

# **Supplementary material**

**Title: Global burden of cerebral small vessel disease with insights from population-based studies with MRI – a systematic review and meta-analysis**

**Section A** Search Strategies used to conduct literature searches

**Section B** Inclusion and exclusion criteria

**Section C** Reviewers involved in the systematic review

**Section D** Details on the influence of MRI parameters on SVD prevalence

**Section E** Supplementary Figures

## Section A Search Strategies used to conduct literature searches

PECO

P = Studies from Global burden of disease regions (n >=400) with MRI

E = Different geographical regions

C = Global average of the cSVD prevalence

O = The prevalence of cSVD lesions on MRI (in %) – including WMH vol/ visual rating; lacune vol/counts; cerebral microbleed; perivascular space, cortical microinfarct, cortical superficial siderosis

Database 1 - OVID MEDLINE

Search date: 31^st^ March 2024

| 1 | exp Cerebral Small Vessel Diseases/ or (Small vessel disease* or SVD or Small vessel cerebrovascular disease* or Small vessel cerebrovascular disorder*).mp. | |
| --- | --- | --- |
| 2 | exp Stroke, Lacunar/ or (subcortical infarct* or rssi* or lacunar infarct* or lacunar stroke* or lacunar lesion* or lacun* or silent brain infarct* or subcortical stroke*).mp. | |
| 3 | exp Leukoaraiosis/ or (white matter hyperintens* or WMH or white matter lesion* or WML or white matter chang* or leukoencephalopath* or white matter disease* or white matter damage*).mp. | |
| 4 | (Perivascular space* or PVS or Virchow?Robin or type 3 lacune or type III lacune or etat crible).mp. | |
| 5 | (cerebral microbleed* or CMB or microbleed or micro?h?emorrhage or dot-like h?emosiderin).mp. | |
| 6 | (micro?infarct or DWI positive lesion).mp. | |
| 7 | superficial siderosis.mp. | |
| 8 | deep intracerebral h?emorrhag*.mp. | |
| 9 | exp dementia, vascular/ | |
| 10 | 1 or 2 or 3 or 4 or 5 or 6 or 7 or 8 or 9 | |
| 11 | exp "Global Burden of Disease"/ or global burden of disease.mp. | |
| 12 | exp Prevalence/ or prevalence.mp. | |
| 13 | exp "Incidence"/ or incidence.mp. | |
| 14 | exp Epidemiology/ or epidemiol*.mp. | |
| 15 | 11 or 12 or 13 or 14 | |
| 16 | exp Case-Control Studies/ or case control.tw. | |
| 17 | exp Cross-Sectional Studies/ or cross-sectional.tw. | |
| 18 | exp Twin Study/ or (twin adj (study or studies)).tw. | |
| 19 | exp Prospective Studies/ or prospective.tw. | |
| 20 | exp Retrospective Studies/ or retrospective.tw. | |
| 21 | exp Cohort Studies/ or (cohort analy$ or (cohort adj (study or studies))).tw. | |
| 22 | population-based.mp. | |
| 23 | 16 or 17 or 18 or 19 or 20 or 21 or 22 | |
| 24 | 10 and 15 and 23 | |
| 25 | Limit 24 to yr="2000 -Current" | |

Database 2 – EMBASE

Search date: 31^st^ March 2024

| 1 | (Small vessel disease* or SVD or Small vessel cerebrovascular disease* or Small vessel cerebrovascular disorder*).mp. |
| --- | --- |
| 2 | exp lacunar stroke/ or (subcortical infarct* or rssi* or lacunar infarct* or lacunar stroke* or lacun* or silent brain infarct* or subcortical stroke* or lacunar lesion*).mp. |
| 3 | exp leukoaraiosis/ or (white matter hyperintens* or WMH or white matter lesion* or WML or white matter chang* or leukoencephalopath* or white matter disease* or white matter damage*).mp. |
| 4 | (Perivascular space* or PVS or Virchow?Robin or type 3 lacune or type III lacune or etat crible).mp. |
| 5 | (cerebral microbleed* or CMB or microbleed or micro?h?emorrhage or dot-like h?emosiderin).mp. |
| 6 | (micro?infarct or DWI positive lesion).mp. |
| 7 | superficial siderosis.mp. |
| 8 | deep intracerebral h?emorrhag*.mp. |
| 9 | exp multiinfarct dementia/ or dementia, vascular.mp. |
| 10 | 1 or 2 or 3 or 4 or 5 or 6 or 7 or 8 or 9 |
| 11 | exp global disease burden/ or global disease burden.mp. |
| 12 | exp prevalence/ or prevalence.mp. |
| 13 | exp incidence/ or incidence.mp. |
| 14 | exp epidemiology/ or epidemiol*.mp. |
| 15 | 11 or 12 or 13 or 14 |
| 16 | population-based.mp. |
| 17 | exp case control study/ or (Case control adj (study or studies)).tw. |
| 18 | exp cross-sectional study/ or (cross sectional adj (study or studies)).tw. |
| 19 | exp twin study/ or (twin adj (study or studies)).tw. |
| 20 | exp prospective study/ or prospective.tw. |
| 21 | exp retrospective study/ or retrospective.tw. |
| 22 | exp cohort analysis/ or (Cohort adj (study or studies)).mp. |
| 23 | 16 or 17 or 18 or 19 or 20 or 21 or 22 |
| 24 | exp “randomized controlled trial (topic)”/ |
| 25 | 23 not 24 |
| 26 | 10 and 15 and 25 |
| 27 | limit 26 to yr="2000 -Current" |

## Section B Inclusion and exclusion criteria

Inclusion:

-  Manuscript published between 1 Jan 2000 and 31 March 2024

-  Original research article published without language restrictions

- Sample size > 400

-  Assess the prevalence (in %) of at least one kind of cSVD lesion on MRI. We will include all variations of the same lesion - WMH (white matter hyperintensities) / WML (white matter lesion) or WMC (white matter change), and include all common methods that they were measured - a) visually rated (common rating scales = FAZEKAS or ARWMC) or b) WMH volume being measured.

- Population-based studies, large community studies or subjects assessed at a hospital with the purpose of health screening only were accepted.

-  Can include cross-sectional or longitudinal study, but only report prevalence at baseline of the longitudinal study

Exclusion criteria:

-   cSVD was diagnosed without the use of MRI (clinical only or with CT only)

-   Study cohorts investigating genetic variants of cSVDs (e.g. CADASIL, CARASIL, etc)

-	Studies that included participants with specific neurological diseases

-	Studies that specifically recruited participants based on the presence of SVD neuroimaging	markers

-   With intervention

-   Non-human studies

-	Postmortem studies

-   Case studies, letters

-   Studies not published in full

## Section C Reviewers involved in the systematic review

Title/ abstract screening

Reviewers: Huijing Zheng (HZ), Jize Wei (JW), Ami Sin Man Li (ASML), Yuan Cai (YC), Vincent Ming Ho Hui (VMHH), Maggie Li Man Lam (MLML), Chin Wai Cheung, King Ngai Chow, Siu Ting Fu, and Kwun Hang Henry Chung

Conflicts resolved by: Bonnie Yin Ka Lam (BYKL), YC

Full-text screening

Reviewers: BYKL, YC, HZ, JW, ASML, VMHH, MLML

Conflicts resolved by: BYKL, YC & VCTM

Data extraction

Reviewers: HZ, JW, ASML, YC, VMHH, MLML, BYKL

Conflicts resolved by: BYKL, YC & VCTM

**Section D** **Details on the influence of MRI parameters on SVD prevalence**

In this study, we assessed how MRI acquisition parameters influence SVD prevalence. In particular, the key parameters examined included: MRI field strength (categorised as < 3T vs 3T), slice thickness (< 2mm vs ≥ 2mm), MRI sequences used for SVD assessment, rating method (Fazekas vs non-Fazekas scales for WMH), and echo time (< 20ms vs ≥ 20ms for CMB assessment). For sequence usage, WMH assessment was categorised as: i) no FLAIR, ii) using only FLAIR; iii) using FLAIR in combination with other sequence(s). The assessment of lacune was categorised as: i) no FLAIR, ii) no T2, iii) using only FLAIR or only T2, and iv) using both FLAIR and T2. Studies that did not clearly report the MRI acquisition parameters in the original article, limited data extraction, and thus, were excluded from the statistical analysis.

We compared studies using field strength ≥ 3T versus < 3T. In the meta-regression, the prevalence of CMB count was higher when the field strength was ≥ 3T compared to that measured when using scanners with lower magnetic field strength. MRI scanners with higher field strength have the advantage of better spatial resolution and higher signal-to-noise ratio, hence, revealing more detailed information of SVD markers at the macroscopic level, and better visualisation of anatomical structures. A few sequences could be used to assess the same marker (e.g. SWI vs T2* GRE vs QSM for assessing CMB), however, there is no consensus on which sequence should be used as the standard for assessment and reporting. In the meta-regression, we observed a trend that using the T2* may have exhibited a lower prevalence of CMB, compared with SWI, although results were only marginally significant. This result is supported by other studies (23, 24). As there is only one study using the QSM, results on whether using the QSM is different from the other sequences (SWI or T2*) warrant further investigation. Slice thickness is another parameter that could influence SVD prevalence (22), such that a thicker slice may overestimate WMH count or CMB count. At the same time, a thicker slice will underestimate WMH volume, as observed in this systematic review and meta-analysis, as well as from a previous study (25).

Other acquisition parameters may also influence prevalence estimates; for instance, WMH volume can differ among different segmentation tools used, especially when the WMH volume in healthy community subjects may only be subtle (e.g., 3 to 6 mL). Due to difficulty in analysing how different software may affect SVD prevalence, this variable was not used in the analysis but reported in the systematic review table instead (see Supplementary Table 1). Another important factor is whether WMH volume was adjusted for intracranial volume. Ideally, WMH should be normalised for intracranial volume (ICV) to adjust for individual head size. While some studies provided only the mean raw WMH volume, some provided the mean WMH ratio (corrected for ICV) without providing the mean raw WMH volume and ICV volume. As more studies provided the mean raw WMH volume (without adjustment), we used the raw WMH volume in the analysis. We performed an additional analysis comparing the forest plot of studies with the mean raw WMH volume compared with the mean WMH ratio (corrected with ICV) and showed that the effect sizes and heterogeneities in both cases were similar. Factors regarding the variability of software or processing tools used were challenging to compare, thus restraining us from deriving further conclusions.

Overall, the meta-regression analyses showed that MRI magnetic field strength, the MRI sequence used, and sequence slice thickness were significant contributors to the variability in the prevalence of SVD. Mean WMH volume and the prevalence of CMB are SVD markers most prone to the variability of MRI acquisition parameters. Our findings emphasised the importance of adopting a harmonised MRI scanning protocol for SVD.

## Section E. Supplementary figures

### **Supplementary Figure 1.1. Forest plot of visual rating moderate-to-severe WMH by** world region (by economic status) and world region (by geography)

### **Supplementary Figure 1.2. Forest plot of visual rating moderate-to-severe WMH by age and study design**

### Supplementary Figure 2.1 Forest plot of WMH volume by world region (by economic status) and world region (by geography)

### Supplementary Figure 2.2 Forest plot of WMH volume by age and study design

### Supplementary Figure 3.1 Forest plot of lacunes by world region (by economic status) and world region (by geography)

### Supplementary Figure 3.2 Forest plot of lacunes by age and study design

### Supplementary Figure 4.1 Forest plot of cerebral microbleeds by world region (by economic status) and world region (by geography)

### Supplementary Figure 4.2 Forest plot of cerebral microbleeds by age and study design

### Supplementary Figure 5.1 Forest plot of moderate-to-severe PVS by world region (by economic status) and world region (by geography)

### Supplementary Figure 5.2 Forest plot of moderate-to-severe PVS by age and study design

### Supplementary Figure 6. Forest plot of the prevalence of cerebral microinfarcts.

### Supplementary Figure 7. Forest plot of the prevalence of cerebral superficial siderosis

### Supplementary Figure 8. Forest plot of the prevalence of the total SVD score

### Supplementary Figure 9. Prevalence stratified by sex

Abbreviations: RD = risk difference; MD = mean difference

=

### Supplementary Figure 10. Forest plot of the prevalence of the WMH ratio (corrected by ICV) and raw WMH volume

### Supplementary Figure 11. Funnel plot of SVD markers
